# Anilinium hydrogen sulfate

**DOI:** 10.1107/S1600536810004782

**Published:** 2010-02-13

**Authors:** Zina Boutobba, Amani Direm, Nourredine Benali-Cherif

**Affiliations:** a Laboratoire des Structures, Propriétés et Interactions Inter-Atomiques., Centre Universitaire Abbes Laghrour, Khenchela 40000, Algeria

## Abstract

The asymmetric unit of the title compound, C_6_H_8_N^+^·HSO_4_^−^, contains two cations and two anions which are linked to each other through N—H⋯O hydrogen bonds, formed by all H atoms covalently bonded to the N atoms. In addition, strong O—H⋯O anion–anion hydrogen-bond inter­actions are also observed.

## Related literature

For hydrogen bonding, see: Zimmerman & Corbin (2000 ▶); Brunsveld *et al.* (2001 ▶); Desiraju (2002 ▶); Desiraju & Steiner (1999 ▶); Steiner (2002 ▶); Etter *et al.* (1990 ▶); Bernstein *et al.* (1995 ▶). For related structures, see: Benali-Cherif, Boussekine *et al.* (2009 ▶); Messai *et al.* (2009 ▶); Benali-Cherif, Falek *et al.* (2009 ▶); Rademeyer (2004 ▶); Jayaraman *et al.* (2002 ▶); Smith *et al.* (2004 ▶); Paixão *et al.* (2000 ▶).
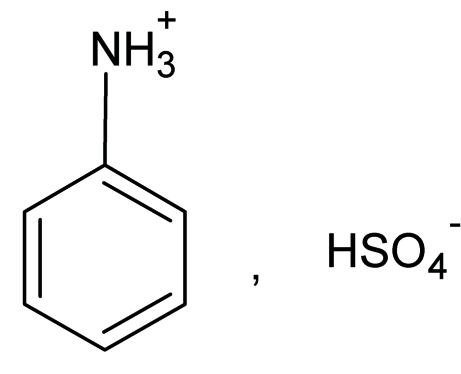


## Experimental

### 

#### Crystal data

C_6_H_8_N^+^·HSO_4_^−^
*M* *_r_* = 191.20
Orthorhombic, 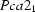

*a* = 14.3201 (2) Å
*b* = 9.0891 (3) Å
*c* = 12.8771 (2) Å
*V* = 1676.04 (7) Å^3^
*Z* = 8
Mo *K*α radiation
μ = 0.36 mm^−1^
*T* = 293 K
0.2 × 0.15 × 0.1 mm

#### Data collection

Nonius KappaCCD diffractometer
16963 measured reflections
4641 independent reflections
3108 reflections with *I* > 2σ(*I*)
*R*_int_ = 0.049

#### Refinement

*R*[*F* ^2^ > 2σ(*F* ^2^)] = 0.041
*wR*(*F* ^2^) = 0.117
*S* = 1.02
4641 reflections
219 parameters
1 restraint
H-atom parameters not refined
Δρ_max_ = 0.35 e Å^−3^
Δρ_min_ = −0.47 e Å^−3^
Absolute structure: Flack (1983 ▶), 2096 Friedel pairs
Flack parameter: 0.08 (9)

### 

Data collection: *KappaCCD Server Software* (Nonius, 1998 ▶); cell refinement: *DENZO* and *SCALEPACK* (Otwinowski & Minor, 1997 ▶); data reduction: *DENZO* and *SCALEPACK*; program(s) used to solve structure: *SIR2004* (Burla *et al.*, 2005 ▶); program(s) used to refine structure: *SHELXL97* (Sheldrick, 2008 ▶); molecular graphics: *ORTEPIII* (Burnett & Johnson, 1996 ▶), *ORTEP-32 for Windows* (Farrugia, 1997 ▶) and *PLATON* (Spek, 2009 ▶); software used to prepare material for publication: *WinGX* publication routines (Farrugia, 1999 ▶).

## Supplementary Material

Crystal structure: contains datablocks I. DOI: 10.1107/S1600536810004782/dn2534sup1.cif

Structure factors: contains datablocks I. DOI: 10.1107/S1600536810004782/dn2534Isup2.hkl

Additional supplementary materials:  crystallographic information; 3D view; checkCIF report

## Figures and Tables

**Table 1:** Hydrogen-bond geometry (Å, °)

*D*—H⋯*A*	*D*—H	H⋯*A*	*D*⋯*A*	*D*—H⋯*A*
N1*A*—H11⋯O1*A*^i^	0.89	1.95	2.821 (2)	167
N1*A*—H22⋯O3*B*	0.89	1.95	2.817 (4)	163
N1*A*—H33⋯O2*B*^iii^	0.89	2.01	2.884 (3)	169
N1*B*—H1⋯O1*B*^ii^	0.89	1.94	2.828 (3)	175
N1*B*—H2⋯O3*A*^ii^	0.89	2.05	2.867 (3)	153
N1*B*—H3⋯O1*A*	0.89	2.58	3.069 (3)	115
N1*B*—H3⋯O2*A*	0.89	2.03	2.916 (3)	175
O4*A*—H4⋯O3*B*	0.82	1.79	2.603 (4)	175
O4*B*—H44⋯O3*A*^i^	0.82	1.84	2.635 (4)	163

Symmetry codes: (i) 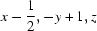; (ii) 
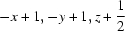
; (iii) 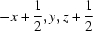.

**Fig. 1.**
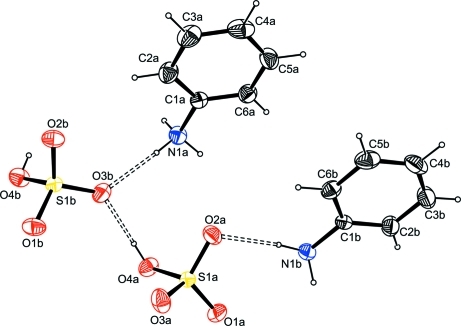
ORTEP view of the asymmetric unit of (I) showing 10% probability displacement ellipsoids.

**Fig. 2.**
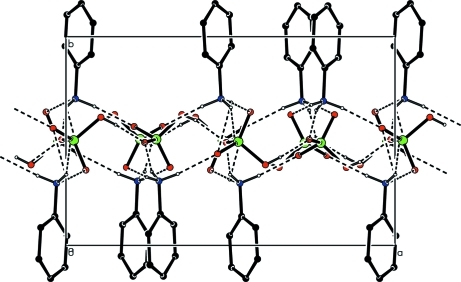
Alternating cationic and anionc layers visualized through the (001) plane.

**Fig. 3.**
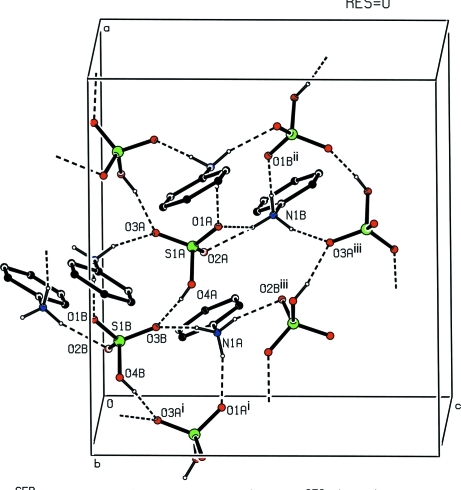
Intermolecular hydrogen bonding patterns running parallel to (bc) plane. H atoms not involved in hydrogen bondings have been omitted for clarity. [Symmetry codes: (i) x-1/2, -y+1, z; (ii) -x+1, -y+1, z+1/2; (iii) -x+1/2, y, z+1/2.

## supplementary crystallographic information

### Comment

The main purpose of this structural study was a determination of the arrangement of the cations and anions which are held together by two-dimensional hydrogen-bond networks.

Hydrogen bonding is one of the most versatile noncovalent forces in supramolecular chemistry and crystal engineering (Zimmerman & Corbin, 2000; Brunsveld *et al.*, 2001; Desiraju, 2002). Therefore, in the past decades assessment of discrete hydrogen bonding patterns had received great attention (Steiner, 2002; Desiraju & Steiner, 1999) because of its widespread occurrence in biological systems.

The aim of this paper is to discuss hydrogen patterns assuring the connection between anilinium and hydrogensulfate entities and to establish their different graph-set motifs (Bernstein *et al.*, 1995).

Bis(anilinium hydrogensulfate) is one of the hybrid compounds, rich in H-bonds (Benali-Cherif, Boussekine, *et al.*, 2009; Messai *et al.*, 2009; Benali-Cherif, Falek, *et al.*, 2009), which could have potential importance in constructing sophisticated assemblies from discrete ionic or molecular building blocks due to the strength and the directionality of hydrogen bonds (Steiner *et al.* 2002, Jayaraman *et al.*, 2002).

Recently, similar structures containing anilinium cations have been reported. Among examples, can be named the folowing ones: anilinium nitrate (Rademeyer, 2004), anilinium picrate (Smith *et al.*, 2004), anilinium hydrogenphosphite and anilinium hydrogenoxalate hemihydrate(Paixão *et al.*, 2000).

The structure of (I) may be described as formed by alternating sheets of cations and anions (Fig. 2) which are held together with four and five-centered N—H···O H-bonds to form C_4_^4^(10) infinite chains running through the c direction. Moreover, strong O—H···O hydrogen bonds observed between bisulfate anions generate C_2_^2^(8) chains in the *a* axis direction. The infinite chains resulting from anion-anion and anion-cation interactions can be described as zigzag layers parallel to the (*ac*) plane (Fig. 3). The crossing of these chains builds up different rings with R_3_^3^(10) and R_5_^4^(16) graph set motifs (Fig. 3) (Etter *et al.*, 1990; Bernstein *et al.*, 1995).

### Experimental

Single crystals of the title compound are prepared by slow evaporation at room temperature of an aqueous solution of aniline and sulfuric acid.

### Refinement

The title compound crystallizes in the centrosymmetric space group P c a 2_1_. All non-H atoms were refined with anisotropic atomic displacement parameters. H atoms were located from Fourier difference maps and treated as riding with C—H = 0.93 Å, N—H = 0.89 Å and O—H = 0.82 Å. Their isotropic displacement parameters were set equal to 1.2Ueq (C) and 1.5Ueq (N, O).

### Crystal data

**Table:**

C_6_H_8_N^+^·HSO_4_^−^	*F*(000) = 800
*M**_r_* = 191.20	*D*_x_ = 1.516 Mg m^−^^3^
Orthorhombic, *P**c**a*2_1_	Mo *K*α radiation, λ = 0.71073 Å
Hall symbol: P 2c -2ac	Cell parameters from 16963 reflections
*a* = 14.3201 (2) Å	θ = 2.7–30.0°
*b* = 9.0891 (3) Å	µ = 0.36 mm^−^^1^
*c* = 12.8771 (2) Å	*T* = 293 K
*V* = 1676.04 (7) Å^3^	Prism, colourless
*Z* = 8	0.2 × 0.15 × 0.1 mm

### Data collection

**Table:**

Nonius KappaCCD diffractometer	3108 reflections with *I* > 2σ(*I*)
Radiation source: fine-focus sealed tube	*R*_int_ = 0.049
graphite	θ_max_ = 30.0°, θ_min_ = 2.7°
ω – θ scans	*h* = −19→17
16963 measured reflections	*k* = −9→12
4641 independent reflections	*l* = −18→16

### Refinement

**Table:**

Refinement on *F*^2^	Secondary atom site location: difference Fourier map
Least-squares matrix: full	Hydrogen site location: inferred from neighbouring sites
*R*[*F*^2^ > 2σ(*F*^2^)] = 0.041	H-atom parameters not refined
*wR*(*F*^2^) = 0.117	*w* = 1/[σ^2^(*F*_o_^2^) + (0.0622*P*)^2^ + 0.1354*P*] where *P* = (*F*_o_^2^ + 2*F*_c_^2^)/3
*S* = 1.02	(Δ/σ)_max_ = 0.001
4641 reflections	Δρ_max_ = 0.35 e Å^−^^3^
219 parameters	Δρ_min_ = −0.47 e Å^−^^3^
1 restraint	Absolute structure: Flack (1983), 2096 Friedel pairs
Primary atom site location: structure-invariant direct methods	Flack parameter: 0.08 (9)

### Special details

**Table:**

Geometry. All esds (except the esd in the dihedral angle between two l.s. planes) are estimated using the full covariance matrix. The cell esds are taken into account individually in the estimation of esds in distances, angles and torsion angles; correlations between esds in cell parameters are only used when they are defined by crystal symmetry. An approximate (isotropic) treatment of cell esds is used for estimating esds involving l.s. planes.
Refinement. Refinement of *F*^2^ against ALL reflections. The weighted *R*-factor wR and goodness of fit *S* are based on *F*^2^, conventional *R*-factors *R* are based on *F*, with *F* set to zero for negative *F*^2^. The threshold expression of *F*^2^ > σ(*F*^2^) is used only for calculating *R*-factors(gt) etc. and is not relevant to the choice of reflections for refinement. *R*-factors based on *F*^2^ are statistically about twice as large as those based on *F*, and *R*- factors based on ALL data will be even larger.

### Fractional atomic coordinates and isotropic or equivalent isotropic displacement parameters (Å^2^)

**Table:**

	*x*	*y*	*z*	*U*_iso_*/*U*_eq_	
N1A	0.21732 (15)	0.3085 (2)	0.3390 (2)	0.0432 (5)	
H22	0.2392	0.3525	0.2823	0.065*	
H33	0.2550	0.3273	0.3924	0.065*	
H11	0.1603	0.3422	0.3530	0.065*	
C1A	0.21307 (19)	0.1496 (3)	0.3217 (3)	0.0363 (7)	
C2A	0.1721 (3)	0.0988 (4)	0.2311 (3)	0.0531 (8)	
H2A	0.1482	0.1641	0.1823	0.064*	
C3A	0.1676 (3)	−0.0512 (4)	0.2153 (3)	0.0626 (10)	
H3A	0.1398	−0.0875	0.1553	0.075*	
C4A	0.2039 (2)	−0.1480 (4)	0.2873 (4)	0.0640 (11)	
H4A	0.2006	−0.2489	0.2760	0.077*	
C5A	0.2449 (3)	−0.0940 (4)	0.3758 (3)	0.0555 (9)	
H5A	0.2698	−0.1588	0.4243	0.067*	
C6A	0.2493 (3)	0.0551 (4)	0.3931 (3)	0.0458 (7)	
H6A	0.2770	0.0912	0.4533	0.055*	
N1B	0.52868 (13)	0.2975 (2)	0.5035 (2)	0.0403 (5)	
H1	0.5861	0.3342	0.4989	0.061*	
H2	0.4989	0.3397	0.5564	0.061*	
H3	0.4978	0.3154	0.4448	0.061*	
C1B	0.53385 (18)	0.1390 (3)	0.5207 (2)	0.0330 (6)	
C2B	0.5767 (2)	0.0855 (4)	0.6081 (3)	0.0498 (8)	
H2B	0.6015	0.1493	0.6573	0.060*	
C3B	0.5824 (3)	−0.0655 (4)	0.6217 (4)	0.0622 (10)	
H3B	0.6104	−0.1037	0.6810	0.075*	
C4B	0.5467 (3)	−0.1590 (4)	0.5479 (4)	0.0622 (11)	
H4B	0.5521	−0.2602	0.5568	0.075*	
C5B	0.5030 (3)	−0.1046 (4)	0.4610 (4)	0.0599 (11)	
H5B	0.4775	−0.1685	0.4122	0.072*	
C6B	0.4973 (3)	0.0456 (4)	0.4468 (3)	0.0453 (7)	
H6B	0.4688	0.0836	0.3877	0.054*	
S1A	0.47642 (6)	0.50293 (6)	0.27815 (5)	0.0354 (3)	
O1A	0.53917 (14)	0.5609 (3)	0.3548 (2)	0.0588 (5)	
O2A	0.43955 (15)	0.3610 (2)	0.30490 (17)	0.0501 (5)	
O3A	0.5129 (3)	0.5072 (3)	0.1721 (3)	0.0610 (11)	
O4A	0.39386 (13)	0.6139 (2)	0.27837 (19)	0.0492 (5)	
H4	0.3495	0.5784	0.2468	0.074*	
S1B	0.22562 (6)	0.50860 (7)	0.06533 (5)	0.0340 (3)	
O1B	0.29288 (13)	0.5696 (2)	−0.00469 (18)	0.0543 (5)	
O2B	0.18378 (13)	0.3739 (2)	0.02858 (18)	0.0477 (5)	
O3B	0.2609 (3)	0.4930 (2)	0.1701 (3)	0.0570 (10)	
O4B	0.14771 (13)	0.6268 (2)	0.0699 (2)	0.0493 (5)	
H44	0.1067	0.6000	0.1100	0.074*	

### Atomic displacement parameters (Å^2^)

**Table:**

	*U*^11^	*U*^22^	*U*^33^	*U*^12^	*U*^13^	*U*^23^
N1A	0.0488 (13)	0.0354 (12)	0.0452 (12)	0.0039 (9)	−0.0019 (9)	−0.0050 (10)
C1A	0.0319 (14)	0.0339 (15)	0.0431 (16)	0.0026 (11)	0.0016 (12)	−0.0062 (12)
C2A	0.0594 (19)	0.0502 (19)	0.0496 (18)	0.0050 (16)	−0.0107 (17)	−0.0079 (17)
C3A	0.069 (2)	0.055 (2)	0.063 (3)	−0.001 (2)	−0.0126 (19)	−0.023 (2)
C4A	0.0542 (19)	0.0386 (19)	0.099 (3)	−0.0010 (14)	0.007 (2)	−0.022 (2)
C5A	0.0558 (18)	0.0426 (17)	0.068 (3)	0.0087 (16)	−0.0002 (19)	0.0097 (17)
C6A	0.0451 (15)	0.045 (2)	0.0471 (18)	−0.0003 (18)	−0.0043 (14)	−0.0026 (16)
N1B	0.0429 (11)	0.0348 (12)	0.0433 (11)	−0.0024 (9)	0.0007 (9)	−0.0008 (10)
C1B	0.0320 (14)	0.0303 (14)	0.0366 (15)	−0.0033 (10)	0.0044 (11)	0.0006 (11)
C2B	0.0532 (18)	0.0478 (18)	0.0483 (17)	0.0025 (15)	−0.0088 (16)	0.0018 (15)
C3B	0.062 (2)	0.058 (3)	0.067 (2)	0.008 (2)	0.003 (2)	0.024 (2)
C4B	0.0561 (19)	0.0345 (18)	0.096 (3)	−0.0007 (15)	0.021 (2)	0.0099 (19)
C5B	0.0511 (18)	0.045 (2)	0.084 (3)	−0.0072 (17)	0.004 (2)	−0.0201 (19)
C6B	0.0410 (15)	0.0461 (19)	0.0487 (19)	−0.0017 (18)	−0.0040 (14)	−0.0100 (17)
S1A	0.0319 (6)	0.0352 (5)	0.0392 (6)	−0.0012 (2)	−0.0005 (5)	0.0038 (2)
O1A	0.0503 (12)	0.0554 (14)	0.0705 (14)	−0.0116 (11)	−0.0226 (10)	0.0051 (12)
O2A	0.0625 (13)	0.0365 (11)	0.0514 (12)	−0.0065 (10)	−0.0036 (10)	0.0067 (8)
O3A	0.058 (2)	0.075 (2)	0.050 (2)	0.0183 (11)	0.0188 (19)	0.0205 (10)
O4A	0.0391 (10)	0.0430 (11)	0.0655 (13)	0.0065 (8)	−0.0047 (9)	−0.0081 (10)
S1B	0.0305 (6)	0.0341 (5)	0.0374 (5)	−0.0020 (2)	0.0013 (4)	−0.0020 (3)
O1B	0.0427 (11)	0.0621 (16)	0.0580 (13)	−0.0112 (10)	0.0141 (9)	0.0022 (12)
O2B	0.0499 (11)	0.0368 (10)	0.0564 (12)	−0.0074 (9)	0.0048 (9)	−0.0099 (9)
O3B	0.0476 (18)	0.073 (2)	0.050 (2)	−0.0086 (10)	−0.0119 (18)	0.0084 (10)
O4B	0.0470 (11)	0.0379 (11)	0.0630 (12)	0.0054 (9)	0.0082 (10)	0.0013 (10)

### Geometric parameters (Å, °)

**Table:**

N1A—C1A	1.462 (4)	C1B—C6B	1.378 (4)
N1A—H22	0.8900	C2B—C3B	1.386 (6)
N1A—H33	0.8900	C2B—H2B	0.9300
N1A—H11	0.8900	C3B—C4B	1.373 (6)
C1A—C6A	1.361 (5)	C3B—H3B	0.9300
C1A—C2A	1.385 (5)	C4B—C5B	1.374 (6)
C2A—C3A	1.380 (5)	C4B—H4B	0.9300
C2A—H2A	0.9300	C5B—C6B	1.380 (4)
C3A—C4A	1.380 (6)	C5B—H5B	0.9300
C3A—H3A	0.9300	C6B—H6B	0.9300
C4A—C5A	1.372 (6)	S1A—O1A	1.435 (2)
C4A—H4A	0.9300	S1A—O2A	1.436 (2)
C5A—C6A	1.375 (4)	S1A—O3A	1.463 (4)
C5A—H5A	0.9300	S1A—O4A	1.554 (2)
C6A—H6A	0.9300	O4A—H4	0.8201
N1B—C1B	1.460 (3)	S1B—O1B	1.431 (2)
N1B—H1	0.8900	S1B—O2B	1.4430 (19)
N1B—H2	0.8900	S1B—O3B	1.448 (4)
N1B—H3	0.8900	S1B—O4B	1.550 (2)
C1B—C2B	1.371 (4)	O4B—H44	0.8200
			
C1A—N1A—H22	109.5	C2B—C1B—N1B	119.8 (3)
C1A—N1A—H33	109.5	C6B—C1B—N1B	119.0 (3)
H22—N1A—H33	109.5	C1B—C2B—C3B	118.8 (3)
C1A—N1A—H11	109.5	C1B—C2B—H2B	120.6
H22—N1A—H11	109.5	C3B—C2B—H2B	120.6
H33—N1A—H11	109.5	C4B—C3B—C2B	120.2 (3)
C6A—C1A—C2A	121.4 (3)	C4B—C3B—H3B	119.9
C6A—C1A—N1A	120.3 (3)	C2B—C3B—H3B	119.9
C2A—C1A—N1A	118.4 (3)	C3B—C4B—C5B	120.7 (4)
C3A—C2A—C1A	118.2 (3)	C3B—C4B—H4B	119.7
C3A—C2A—H2A	120.9	C5B—C4B—H4B	119.7
C1A—C2A—H2A	120.9	C4B—C5B—C6B	119.4 (3)
C2A—C3A—C4A	120.9 (3)	C4B—C5B—H5B	120.3
C2A—C3A—H3A	119.6	C6B—C5B—H5B	120.3
C4A—C3A—H3A	119.6	C1B—C6B—C5B	119.7 (3)
C5A—C4A—C3A	119.4 (3)	C1B—C6B—H6B	120.2
C5A—C4A—H4A	120.3	C5B—C6B—H6B	120.2
C3A—C4A—H4A	120.3	O1A—S1A—O2A	113.28 (16)
C4A—C5A—C6A	120.5 (3)	O1A—S1A—O3A	114.1 (2)
C4A—C5A—H5A	119.8	O2A—S1A—O3A	112.27 (15)
C6A—C5A—H5A	119.8	O1A—S1A—O4A	103.72 (14)
C1A—C6A—C5A	119.6 (3)	O2A—S1A—O4A	107.62 (12)
C1A—C6A—H6A	120.2	O3A—S1A—O4A	104.85 (16)
C5A—C6A—H6A	120.2	S1A—O4A—H4	109.5
C1B—N1B—H1	109.5	O1B—S1B—O2B	113.68 (16)
C1B—N1B—H2	109.5	O1B—S1B—O3B	113.0 (2)
H1—N1B—H2	109.5	O2B—S1B—O3B	111.55 (14)
C1B—N1B—H3	109.5	O1B—S1B—O4B	103.86 (13)
H1—N1B—H3	109.5	O2B—S1B—O4B	107.56 (12)
H2—N1B—H3	109.5	O3B—S1B—O4B	106.47 (17)
C2B—C1B—C6B	121.2 (3)	S1B—O4B—H44	109.5
			
C6A—C1A—C2A—C3A	0.9 (5)	C6B—C1B—C2B—C3B	−0.4 (4)
N1A—C1A—C2A—C3A	−179.6 (3)	N1B—C1B—C2B—C3B	−178.8 (3)
C1A—C2A—C3A—C4A	−0.7 (5)	C1B—C2B—C3B—C4B	0.9 (4)
C2A—C3A—C4A—C5A	0.0 (6)	C2B—C3B—C4B—C5B	−1.6 (5)
C3A—C4A—C5A—C6A	0.4 (6)	C3B—C4B—C5B—C6B	1.7 (6)
C2A—C1A—C6A—C5A	−0.4 (5)	C2B—C1B—C6B—C5B	0.5 (5)
N1A—C1A—C6A—C5A	−180.0 (3)	N1B—C1B—C6B—C5B	178.9 (3)
C4A—C5A—C6A—C1A	−0.2 (5)	C4B—C5B—C6B—C1B	−1.1 (5)

### Hydrogen-bond geometry (Å, °)

**Table:**

*D*—H···*A*	*D*—H	H···*A*	*D*···*A*	*D*—H···*A*
N1A—H11···O1A^i^	0.89	1.95	2.821 (2)	167
N1B—H2···O3A^ii^	0.89	2.05	2.867 (3)	153
N1A—H33···O2B^iii^	0.89	2.01	2.884 (3)	169
N1B—H3···O1A	0.89	2.58	3.069 (3)	115
N1B—H3···O2A	0.89	2.03	2.916 (3)	175
N1A—H22···O3B	0.89	1.95	2.817 (4)	163
O4A—H4···O3B	0.82	1.79	2.603 (4)	175
O4B—H44···O3A^i^	0.82	1.84	2.635 (4)	163
N1B—H1···O1B^ii^	0.89	1.94	2.828 (3)	175

Symmetry codes: (i) *x*−1/2, −*y*+1, *z*; (ii) −*x*+1, −*y*+1, *z*+1/2; (iii) −*x*+1/2, *y*, *z*+1/2.
